# Nordic Walking as a Non-Pharmacological Intervention for Chronic Pain and Fatigue: Systematic Review

**DOI:** 10.3390/healthcare12121167

**Published:** 2024-06-08

**Authors:** Daniel González-Devesa, Silvia Varela, Miguel Adriano Sanchez-Lastra, Carlos Ayán

**Affiliations:** 1 Well-Move Research Group, Galicia Sur Health Research Institute (IIS Galicia Sur), SERGAS-UVIGO, 36310 Vigo, Spain; danidevesa4@gmail.com (D.G.-D.); misanchez@uvigo.es (M.A.S.-L.); cayan@uvigo.es (C.A.); 2 Department of Special Didactics, University of Vigo, 36005 Pontevedra, Spain

**Keywords:** Nordic walking, pain, fatigue, randomized controlled trials

## Abstract

Objective: We aimed to analyze and summarize the available scientific evidence on the benefits of Nordic walking for people with chronic pain and fatigue. Literature Survey: This systematic review adhered to PRISMA guidelines and conducted a comprehensive search across five databases using the PICO strategy. Methodology: Inclusion criteria encompassed randomized trials evaluating Nordic walking for pain and fatigue. Two authors independently screened studies, extracted data, and assessed methodological quality using the PEDro scale. Synthesis: A total of 14 studies were included, with sample sizes ranging from 20 to 136 participants. The methodological quality of the included studies varied from fair (five studies) to good (nine studies). The interventions consisted of supervised Nordic walking sessions lasting 6 to 24 weeks, with a frequency of 2 to 4 days per week and duration of 25 to 75 min. The results of this review suggest that Nordic walking had beneficial effects in six of the eight studies that analyzed participant fatigue. However, Nordic walking did not show greater beneficial effects on fatigue than walking (two studies) or than not performing physical activity (one study). Additionally, six of the nine studies that examined the effects of Nordic walking on participants’ perceptions of pain showed beneficial results. However, five studies that compared Nordic walking with control groups did not find any significant inter-group differences on pain. Conclusions: Based on our findings, Nordic walking exercise programs provide a potentially efficient method for alleviating pain and fatigue in people with chronic conditions. Its straightforwardness and ease of learning make it accessible to a broad spectrum of participants, which can result in higher adherence rates and lasting positive effects.

## 1. Introduction

Pain and fatigue are among the most common self-reported symptomatic conditions presented in primary care [1]. Chronic pain is a medical condition that affects millions of people worldwide and imposes a considerable economic cost due to an increase in healthcare expenses and loss of productivity [2]. Similarly, self-reported fatigue is also a prevalent symptom that impairs quality of life and carries a significant health-related economic cost per patient [3].

Pain and fatigue are symptoms strongly related to each other [4]. Biological mechanisms that explain this association include systemic inflammation, which originated from a higher level of pro-inflammatory cytokines involved in tissue repair [5], a reduction in brain-derived neurotrophic factor [6], and lower dopamine levels [7]. Both pain and fatigue are associated with a poorer quality of life and co-occurring symptoms, leading physicians to frequently advise on how to manage both conditions [8].

Since pharmacological therapies have limited efficacy, non-pharmacological strategies are strongly advised for the management of pain and fatigue, among which exercise is highly recommended [9,10]. Most exercise programs implemented for pain and fatigue management are supervised and typically performed several days per week at sports facilities [11,12]. This setup requires transportation and financial resources, which have been previously identified as barriers to exercise for individuals experiencing pain [13] or fatigue [14]. Therefore, other more feasible exercise options warrant further exploration in these populations.

This could be the case for Nordic walking (NW), an exercise modality that does not require a high economic investment in material resources and can be performed autonomously after a short period of learning [15]. The practice of NW has proven feasible for populations where pain [16] and fatigue [17] are predominant symptoms. Therefore, health and rehabilitation professionals should consider NW as a viable option for managing pain or fatigue.

However, before recommending NW as a therapeutic approach, rehabilitation professionals should be informed with quality and up-to-date information regarding its potential benefits for pain and fatigue management, as well as its role in achieving current international physical activity guidelines. Achieving this goal can be facilitated by conducting systematic reviews that synthesize and summarize the existing scientific evidence on the subject. To the best of the authors’ knowledge, no systematic review focusing on the effects of NW for managing pain and fatigue has been published to date. In light of this, the purpose of this study is to conduct a systematic review to identify and critically analyze the best available evidence concerning the utility of prescribing NW for managing pain and fatigue.

## 2. Materials and Methods

This systematic review was conducted following the Preferred Reporting Items for Systematic Reviews and Meta-Analyses (PRISMA) guidelines [18]. This review was registered with the Open Science Framework (OSF), https://doi.org/10.17605/OSF.IO/P5YKE.

### 2.1. Literature Search Strategy

A systematic search was conducted across three electronic databases—PubMed, Scopus, and SportDiscus—from their inception dates up to March 2024. Following the recommendations of the Cochrane Handbook for Systematic Reviews of Interventions to broaden the systematic search [19], we applied the population, intervention, comparison, and outcome (PICO) strategy, including only search terms regarding the population (people with pain and/or fatigue) and intervention (Nordic walking), in a combination of standardized MeSH and free-text terms. Therefore, the following search terms, Boolean operators, and combinations were used: (“Nordic Walking” OR “Pole Walking”) AND (fatigue OR pain OR “pain perception”). The reference list of the included studies and relevant systematic reviews was also examined for potentially eligible studies.

### 2.2. Inclusion and Exclusion Criteria

Intervention studies eligible for the review met the following inclusion criteria: (1) randomized controlled trial (RCT) design; (2) NW-based interventions in at least one of the groups; and (3) included pain, pain perception, and/or fatigue outcomes, assessed by means of specific measurement tools. Studies were excluded if they did not meet the inclusion criteria, the NW was combined with other therapies, or if they were not written in languages in which the authors are fluent, specifically English, Spanish, or Portuguese.

### 2.3. Study Selection

Two authors (D.G. and S.V.) screened the titles and abstracts of the identified studies for eligibility, independently reviewed the full text of the potentially eligible studies, selected the works that met the inclusion criteria, and compared the results to reach an agreement. If it was unclear whether the study met the selection criteria, advice was sought from a third author (C.A.), and a consensus was reached.

### 2.4. Data Extraction

Data from the included studies were extracted from the original reports by two researchers and reviewed by a third investigator. The data extraction form included author’s names, year of publication, sample size, participant’s characteristics, NW intervention and control characteristics, adverse events arising from physical exercise and adherence, dropouts and completion rates, and main results. We also extracted available data on the assessment methods of pain and fatigue.

### 2.5. Quality Appraisal

The methodological quality of the selected RCTs was directly retrieved from the Physiotherapy Evidence Database (PEDro) [20]. Studies were categorized as excellent (score of 9–10), good (6–8), fair (4–5), or poor quality (≤3) [21].

## 3. Results

### 3.1. Design and Samples

**Figure 1:**
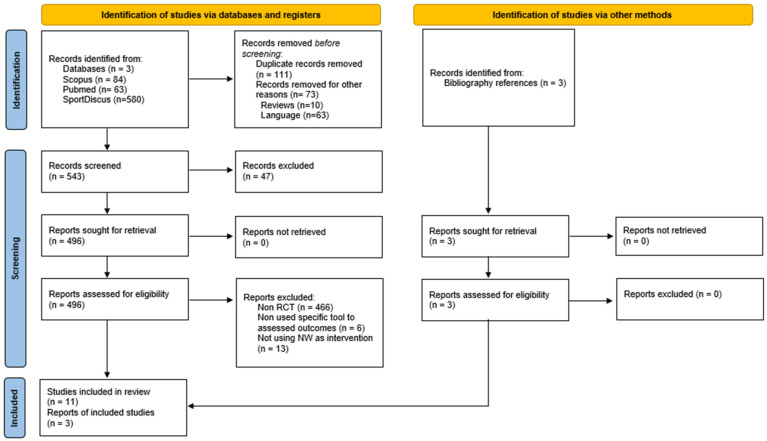
PRISMA 2020 flow diagram for new systematic reviews, which included searches of databases and registers only.

Out of the initial pool of 727 records, 496 were selected for a full-text assessment, and initially 11 studies were chosen for the review. After conducting a citation search, an additional three new records meeting the inclusion criteria were identified, resulting in a total of 14 RCTs ultimately included in the analysis (Figure 1). All the studies were published between 2003 and 2023.

The total sample size from all studies was 692 participants. Sample sizes in the reviewed RCTs ranged from 20 to 136 participants, with an age range spanning from 22 to 92 years. The characteristics of the participating populations were heterogeneous in terms of health status. Thus, populations with peripheral vascular disease [22,23], Parkinson’s disease [24,25,26], aromatase inhibitor-associated arthralgia [27], chronic back pain [15], fibromyalgia [28,29], non-specific neck and shoulder pain [30], multiple sclerosis [31], in geriatric rehabilitation [32], office workers [33], and adults after COVID-19 infection [34] were investigated. Detailed information on the characteristics of the included trials is presented in Table 1.

### 3.2. Dropouts and Adverse Events

A total of 68 dropouts were observed across the ten studies [15,22,23,26,27,29,30,31,32,33] that provided information on this matter, with 39 of them occurring in the exercise group. The primary reasons for dropouts included medical reasons, discontinuing the intervention, and/or personal reasons. None of the reviewed studies reported adverse effects derived from the training programs.

### 3.3. Quality Appraisal

The methodological quality of the RCTs was considered fair in five studies [23,24,27,28,30] and good in nine studies [15,22,25,26,29,31,32,33,34]. A full description of the quality analysis was also provided (see Table 2).

None of the studies blinded either the therapist or participants. In only five studies the assessors were blinded. Only in five studies all participants for whom outcome measures were available received the treatment or control condition as allocated or, where this was not the case, data for at least one key outcome were analyzed by “intention to treat” (section 8 of the PEDro scale).

### 3.4. General Characteristics of the Interventions

The main characteristics of the NW interventions and strategies implemented for the control groups are shown in Table 1. In seven investigations, the control groups did not increase their physical activity levels [15,22,24,27,30,32,33,34].

In four studies, NW was compared against walking without poles [23,26,28,29], while one investigation included a second comparison group performing unsupervised NW [15]. Two studies compared NW vs. another exercise modality such as strengthening [30] and aerobic training on a cycloergometer and treadmill [31]. In the work conducted by Deepa et al. [25], the control group performed tele-rehabilitation exercises focusing on cardiovascular health, balance, and strengthening.

Most exercise programs were supervised by a health professional or NW instructor for the entire duration of the intervention [15,22,24,25,26,27,28,29,30,31,32,33,34]. One of the studies included a period of unsupervised exercise after several weeks of initial learning [27], and in two investigations, the NW program was performed autonomously with indications provided by the instructors [23,25]. Additionally, nine studies reported that NW was performed in groups and outdoors, prioritizing natural environments (e.g., forests or gardens) [15,24,25,26,27,29,31,33,34].

The duration of the interventions ranged from 6 to 24 weeks. These included sessions of 25 to 75 min in length, which were performed with a frequency of 2 to 4 days per week. Training intensity was specified in most RCTs, with the exception of four of these [15,26,27,28]. Four of the interventions reviewed established mean reserve heart rate (HR) values ranging from 40% to 80% controlled using a heart rate monitor [24,25,31,33]. Two of the studies [22,34] used the maximum HR (between 60% and 80%), and in two studies the Borg perceived exertion scale (RPE) [29,30]. In addition, three studies indicated intensity using subjective descriptors: “participants” comfortable and habitual pace” [32], “normal pace” [23], and “desired speed” [15].

Of those reviewed, three studies [15,28,29,30] conducted follow-up evaluations. In the study by Hartvigsen et al. [15], the intervention was evaluated at the end of the study duration (8 weeks), with a follow-up test at 6 months from the beginning of the intervention and another one at 12 months. In the case of Bjersing et al. [28], a single follow-up was performed at 30 weeks. Mannerkorpi et al. [29] conducted a follow-up test 6 months after the initial evaluation. Saeterbakken et al. [30] tested participants after the intervention (10 weeks) and at a follow-up test 10 weeks post-intervention.

### 3.5. Main Outcomes

Significant changes (*p* < 0.05) were found between pre- and post-scores in the NW experimental groups for pain and/or fatigue outcomes in eleven of the fourteen investigations analyzed [15,22,24,25,26,27,29,30,31,33,34]. The measuring instruments used for these two variables are detailed in the following subsections and in Table 1.

#### 3.5.1. Pain

Nine studies looked at the effect of NW on participants’ pain perception [15,22,23,27,28,29,30,32,33]. Of these, six reported a significant intra-group reduction in pain [15,22,27,29,30,33].

Mannerkorpi et al. [29] reported a significant reduction in pain within the NW group when compared to the walking group after 15-week intervention in a woman with fibromyalgia. In this regard, Kocur et al. [33] showed that 12-week NW reduces tenderness in infraspinatus, brachioradial, and latissimus dorsi muscles compared to an inactive control group in postmenopausal female office workers. However, other studies that compared NW with control groups did not find any significant inter-group differences on pain [15,22,23,28,30].

The two investigations that included normal walking yielded mixed results [22,23]. Collins et al. [22] reported that 24-week NW intervention led to significant changes, but no effect of walking on pain was observed. On the contrary, Spafford et al. [23] did not report that either NW or walking had any significant effect after a 12-week intervention.

In the study conducted by Saeterbakken et al. [30], the effects were maintained in the NW group after the 10-week follow-up compared to baseline, but differences between the NW and the control groups remained unchanged in participants with low non-specific neck and shoulder pain. Hartvigsen et al. [15] observed no significant inter-group differences after the follow-up; however, pain was lower at the 26-week follow-up in the supervised NW group compared to baseline. Bjersing et al. [28] and Mannerkorpi et al. [29] observed no differences at follow-up on pain.

#### 3.5.2. Fatigue

Eight studies analyzed fatigue outcomes following NW interventions [2,3,4,5,6,7,8,9,10,11,12,13,14,15,16,17,18,19,20,21,22,23,24,25,26,29,31,34]. Six of them reported significant positive changes in the NW group after the intervention [22,24,25,26,31,34]. Furthermore, Mannerkorpi et al. [29] observed significant positive changes after the 26-week follow-up for NW and control groups in women with fibromyalgia.

Five studies analyzed inter-group differences. Cugusi et al. [24] and Deepa et al. [25] reported that NW had superior effects to usual care or to combined exercise training, respectively. In the remaining three investigations, NW did not show greater beneficial effects on fatigue than walking [23,29] or than not performing physical activity [22] after interventions.

## 4. Discussion

This review aimed to provide insights into the effectiveness of prescribing Nordic walking as a rehabilitation method for managing pain and fatigue. To analyze the highest quality of evidence available, we included only RCT, which showed mostly good and fair methodological quality, strengthening the consistency of our findings. The synthesis of evidence and program characteristics presented in this review may assist health professionals in the design and implementation of NW programs for this population.

Exercise has been shown to be effective for reducing pain [35], a finding that is in agreement with previous research. In a systematic review in which 17 studies were pooled and meta-analyzed, it was found that walking had small to moderate positive effects on chronic musculoskeletal pain [36]. Exercise-induced hypoalgesia has also been noted following the completion of individual aerobic exercise sessions [37].

The positive effect of exercise on pain is usually explained through the opioid theory hypothesis, which states that activation of the endogenous opioid system during exercise may be responsible for exercise-induced hypoalgesia [38]. In accordance with this, six of the reviewed studies provided scientific evidence supporting the prescription of NW practice. However, the findings of the present review cast doubt on the efficacy of NW for managing this symptom. This assumption arises after determining that out of the nine studies that provided information on this outcome, three of them did not report any significant changes derived from its practice. In addition, three investigations did not show superior effects of NW compared to control groups that followed usual care. The practice of NW was neither more effective than strength training, which is an interesting finding. Strength training has also been shown to be effective in alleviating pain for various conditions, such as fibromyalgia (using weight machines) [39] and chronic back pain (through core-based exercises) [40]. Our findings support previous results indicating that strengthening is equally beneficial to aerobic exercise for relieving pain [41,42].

Considering these findings, it is plausible to hypothesize that NW can positively influence the ability to cope with pain, but evidence regarding its effects at the level at which pain is perceived (pain threshold) is not conclusive as previously observed in other exercise modalities [43].

Regarding the impact of NW on fatigue, results are also inconsistent. Most of the proposed interventions reported positive changes in this outcome among participants who engaged in this exercise modality. These results are in accordance with other investigations that have proposed aerobic training as a successful exercise modality for fatigue management [44,45]. Nevertheless, three investigations did not confirm that NW had superior effects compared to the control groups. In this regard, studies comparing the impact of various exercise modalities on fatigue have concluded that aerobic activity should be combined with other types of training to maximize its potential benefits [46,47]. Putting all these findings together, it can be hypothesized that integrating NW practice as part of a combined exercise training program could be an interesting approach to maximize its positive impact on fatigue.

From the reviewed studies, three interesting findings warrant further discussion. Firstly, the investigations that assessed the impact of NW in both fatigue and pain showed a similar effect of this therapy on these symptoms, suggesting that pain and fatigue reductions seemed to be positively associated with each other, as previously observed [48]. This result may be based on the fact that the underlying mechanisms explaining the effects of physical exercise on pain and fatigue (e.g., pro-inflammatory cytokines, brain-derived neurotrophic factor) are common to both outcomes. Secondly, according to the observed results, it cannot be concluded that NW has superior effects to walking without poles. This finding is in agreement with previous observations indicating that the advantages of NW over free walking are unclear [49,50]. Finally, the impact of the interventions was not strongly related to whether they were performed with or without supervision. Therefore, while session supervision appears to enhance the beneficial effects, potentially through increased adherence rates, NW appears to be easy to learn and perform autonomously. Its benefits for reducing pain and fatigue may also be achieved under unsupervised practice. This increases the appeal of this type of exercise for settings with fewer human and economic resources. In any event, factors such as physical activity and fitness levels, as well as the state of the injury or pain condition, influence whether exercise promotes analgesia or increases pain. Therefore, it is plausible to think that the effects of NW interventions may also depend on the source of pain and fatigue [5,51].

This systematic review presents some limitations that must be considered. Firstly, the health conditions included in the different studies of this review were diverse, which limits the comparability, generalizability, and extrapolations of the present findings. Secondly, most of the studies included small sample sizes, and the heterogeneous interventions and control groups limited the possibility of conducting a meta-analysis. Thirdly, few studies compared the impact of NW versus other exercise interventions. Therefore, it was not possible to conduct a detailed analysis to determine whether its practice is superior to other training programs. Finally, there are limitations related to the fact that we did not review the gray literature and to publication bias, which may have influenced the results shown here.

## 5. Conclusions

NW exercise programs offer a potentially effective approach for reducing pain and fatigue in individuals living with chronic conditions. The simplicity and learnability of this exercise make it accessible to a wide range of participants, potentially leading to increased adherence rates and long-term benefits.

## Figures and Tables

**Table 1:** Characteristics of the studies included in this review.

Authors (Year)	Participants	Intervention	Variables (Test)	Adverse Effects/Completion Rate	Results
Acar et al. (2023) [34]	**Initial/final sample size**: 30/30 adults after COVID-19 infection **Distribution** (*n*; % W; median age ± range): - **IG**: n = 15; 80% W; 24 (22–49) - **CG**: n = 15; 66.66% W; 24 (22–50)	**Duration**: 6 weeks **IG**: Exercise: NW Volume: 25–55 min/session Intensity: 60–80% HR_max_, 4–6 RPE (modified) Frequency: 3 sessions/week CG: Did not performed any exercise program	- Fatigue (FSS)	**Adverse effects**: NR **Completion rate**: 100% **Dropouts**: NO **Reasons for dropout/exclusion**: NO	**Intra-group** (*p* < 0.05) ↓ Fatigue in IG after intervention **Inter-group** (*p* < 0.05) NR
Bjersing et al. (2012) [28]	**Initial/final sample size**: 49/49 Woman with fibromyalgia **Distribution** (*n*; % W; mean age/range): - **IG**: n = 26; 100% W; 52 (48–56) - **CG**: n = 23; 100% W; 52 (48–56)	**Duration**: 15 weeks + 30 weeks follow-up **IG**: Exercise: NW Volume: 40–45 min/session Intensity: NR Frequency: 2 sessions/week **CG**: Supervised low intensity walking.	- Pain threshold (KPa Algometer) - Pain experienced during the last week (FIQ visual scale)	**Adverse effects**: NR **Completion rate**: 100% **Dropouts**: NO **Reasons for dropout/exclusion**: NO	**Intra-group** (*p* < 0.05) NO **Inter-group** (*p* < 0.05) NO
Collins et al. (2003) [22]	**Initial/final sample size**: 52/46 with peripheral artery disease **Distribution** (*n*; % W; mean age ± SD): - IG + vitamin E *: *n* = 13; 7.7% W; 67.5 ± 5.8 - IG + placebo *: *n* = 14; 0% W; 63.6 ± 7.8 - CG + vitamin E *: *n* = 13; 0% W; 67.2 ± 9.4 - CG + placebo *: *n* = 12; 0% W; 70.2 ± 8.3 * Vitamin E supplement (400 international units) or a placebo corresponding to vitamin E was provided to each of IG or CG.	**Duration**: 24 weeks **IG (with/without vitamin E)**: Exercise: NW Volume: 45–60 min/session Intensity: 70–80% HR_max_ Frequency: 3 sessions/week. **CG (with/without vitamin E)**: Without exercise.	- Pain (Rating of Perceived Pain, Borg scale) - Fatigue (RPE, Borg scale)	**Adverse effects**: NR **Completion rate**: 88.46% **Dropouts**: IG + vitamin E = 1 IG + placebo = 3 CG + vitamin E = 1 CG + placebo = 1 **Reasons for dropout/exclusion**: - 4 withdrew for medical reasons - 2 for personal reasons.	**Intra-group** (*p* < 0.05) ↓ Pain in IG at 24 weeks ↓ Fatigue in IG at 24 weeks **Inter-group** (*p* < 0.05) NO
Cugusi et al. (2015) [24]	**Initial/final sample size**: 20/20 with Parkinson’s disease **Distribution** (*n*; % W; mean age ± SD): - **IG**: n = 10; 20% W; 68.1 ± 8.7 - **CG**: n = 10; 20% W; 66.6 ± 7.3	**Duration**: 12 weeks **IG**: Exercise: NW Volume: 60 min/session Intensity: 60–80% HR reserve Frequency: 2 sessions/week **CG**: Conventional care for Parkinson’s disease.	- Fatigue (PFS-16) - Fatigue (NMSS)	**Adverse effects**: NR **Completion rate**: 100% **Dropouts**: NO **Reasons for dropout/exclusion**: NO	**Intra-group** (*p* < 0.05) ↓ Fatigue (PFS-16) in IG at 12 weeks ↓ Fatigue (NMSS) in IG at 12 weeks **Inter-group** (*p* < 0.05) < PFS-16 score in IG compared with CG at 12 weeks. < NMSS score in IG compared with CG at 12 weeks.
Deepa et al. (2023) [25]	**Initial/final sample size**: 44/44 with Parkinson’s disease **Distribution** (*n*; % W; mean age ± SD): - **IG**: *n* = 22; 100% W; 53.6 ± 5.1 - **CG**: *n* = 22; 100% W; 52.2 ± 6.3	**Duration**: 9 weeks **Volume**: 40 min/session **Frequency**: 4 sessions/week **IG**: Exercise: Tele-rehabilitation NW Intensity: 60–80% HR reserve **CG**: Exercise: Tele-rehabilitation cardiovascular, balance and strengthening exercises. Intensity: NR	- Fatigue (FSS)	**Adverse effects**: NR **Completion rate**: 100% **Dropouts**: NO **Reasons for dropout/exclusion**: NO	**Intra-group** (*p* < 0.05) ↓ Fatigue in IG at 9 weeks ↓ Fatigue in CG at 9 weeks **Inter-group** (*p* < 0.05) < FFS score in CG compared with IG at 9 weeks.
Fields et al. (2016) [27]	**Initial/final sample size**: 40/36 Women, with surgery for breast cancer, with arthralgia associated with aromatase inhibitors. **Distribution** (*n*; % W; mean age ± SD): - **IG**: *n* = 20; 100% W; 60 ± 8 - **CG**: *n* = 20; 100% W; 66 ± 7	**Duration**: 12 weeks (6 supervised weeks + 6 autonomous weeks) **IG**: Exercise: NW Volume: - Supervised sessions: 60 min - Autonomous sessions: 30 min Intensity: NR Frequency: - Supervised weeks: 4 supervised + 1 autonomous sessions/week - Autonomous weeks: 4 sessions/week **CG**: They were prescribed neither the NW intervention nor any other type of physical exercise. They did receive a brochure on healthy lifestyle and were contacted biweekly to find out the state of health/fitness habits.	- Pain (BPI-SF)	**Adverse effects**: NR **Completion rate**: 90% **Dropouts**: - IG: *n* = 4 **Reasons for dropout/exclusion**: - 2 participants dropped out before the intervention started - 1 for work commitments - 1 for sudden grief	**Intra-group** ↓ Pain in IG and CG at 6 and 12 weeks **Inter-group** NR
Figueiredo et al. (2013) [32]	**Initial/final sample size**: 30/26 older adults **Distribution** (*n*; % W; mean age ± SD): - **IG**: *n* = 14; 57% W; 78 ± 7 - **CG**: *n* = 16; 56% W; 78 ± 7	**Duration**: 6 weeks **Volume**: 20 min **Intensity**: Comfortable and usual pace **Frequency**: 2 sessions / week **IG**: Exercise: NW **CG**: Walking without poles supervised by an instructor.	- Pain (VAS 0–100)	**Adverse effects**: NR **Completion rate**: 86.67% **Dropouts**: - IG: *n* = 1 - CG: *n* = 3 **Reasons for dropout/exclusion**: - 3 worsened by performing the interventions - 1 passed away	**Intra-group** (*p* < 0.05) NO **Inter-group** (*p* < 0.05) NR
Granziera et al. (2020) [26]	**Initial/final sample size**: 37/32 with Parkinson’s disease **Distribution** (*n*; % W; mean age ± SD): - **IG**: *n* = 16; 37,5% W; 68.8 ± 10.2 - **CG**: *n* = 16; 32,2% W; 68.3 ± 6.2	**Duration**: 8 weeks **IG**: Exercise: NW Volume: 75 min/session Intensity: NR Frequency: 2 sessions/week **CG**: Walking without poles (outdoors).	- -Fatigue (PFS-16) - Fatigue (NMSS)	**Adverse effects**: NR **Completion rate**: 86.49% **Dropouts**: IG: *n* = 2 CG: *n* = 3 **Reasons for dropout/exclusion**: - 4 Lack of adherence - 1 falls/ traumatic fractures before the start of intervention.	**Intra-group** (*p* < 0.05) ↓ Fatigue (PFS-16) in IG and CG at 8 weeks ↓ Fatigue (NMSS) in IG and CG at 8 weeks **Inter-group** (*p* < 0.05) NR
Hartvigsen et al. (2010) [15]	**Initial/final sample size**: 136/126 with low back and/or leg pain of greater than eight weeks duration **Distribution** (*n*; % W; mean age ± SD): - **IG**: *n* = 45; 77.5% W; 49.2 ± 11.1 - **CG** A: *n* = 46; 69.1% W; 45.4 ± 10.8 - **CG B**: *n* = 45; 68.2% W; 45.5 ± 11	**Duration**: 8 weeks + 52 weeks follow-up **IG**: Exercise: NW supervised by an instructor. Volume: ~45 min/session Intensity: NR Frequency: 2 sessions/week **CG** A: NW not supervised. A 60-min NW session and permission to do as much NW as they would like for the next 8 weeks providing poles. CG B: They received information about leading an active lifestyle and maintaining functionality in daily life.	- Pain (LBPRS)	**Adverse effects**: NR **Completion rate**: 92.65% **Dropouts**:IG: *n* = 5 CG A: *n* = 4 CG B: *n* = 1 **Reasons for dropout/exclusion**: 5 Inability to adhere to the intervention program.	**Intra-group** (*p* < 0.05) ↓ Pain in IG and CG A at 8 weeks ↓ Pain in IG at 26 weeks follow-up. **Inter-group** (*p* < 0.05) NO
Kocur et al. (2017) [33]	**Initial/final sample size**: 44/32 postmenopausal female office workers **Distribution** (*n*; % W; mean age ± SD): - **IG**: *n* = 22; 100% W; 54.5 ± 3.7 - **CG**: *n* = 22; 100% W; 56.7 ± 2.9	**Duration**: 12 weeks **IG**: Exercise: NW Volume: 60 min/session. Intensity: 40–70% HR reserve. Frequency: 3 sessions/week **CG**: They didn’t change their exercise habits.	- Pain threshold * (Algometer, kg/cm^2^) * **Measured in**: Trapezius, infraspinatus, brachioradial, pec. maj., latissimus dorsi and middle trapezius muscles.	**Adverse effects**: NR **Completion rate**: 88.64% **Dropouts**: IG: *n* = 2 CG: *n* = 3 **Reasons for dropout/exclusion**: - IG: Deterioration of health, personal reasons. - CG: personal reasons, starting to do physical activity	**Intra-group** (*p* < 0.05) ↑ Pain threshold (infraspinatus, latissimus dorsi and middle trapezius muscles) in IG at 12 weeks **Inter-group** (*p* < 0.05) < Pain threshold (infraspinatus, brachioradial, latissimus dorsi muscles) in IG compared with CG at 12 weeks.
Mannerkorpi et al. (2010) [29]	**Initial/final sample size**: 67/58 Woman with fibromyalgia **Distribution** (*n*; % W; mean age ± SD): - **IG**: *n* = 34; 100% W; 48 ± 7.8 - **CG**: *n* = 33; 100% W; 50 ± 7.6	**Duration**: 15 weeks + 26 weeks follow-up **Volume**: 20–30 min/session. **IG**: Exercise: NW Intensity: Moderate-to-high. 9–15 RPE Frequency: 2 sessions/week **CG**: Exercise: Walking Intensity: Low. 9–11 RPE intensity Frequency: 1 session/week	- Pain (FIQ pain subscale) - Fatigue (MFI)	**Adverse effects**: NR **Completion rate**: 86.57% **Dropouts**: *n* = 9 **Reasons for dropout/exclusion**: NR	**Intra-group** (*p* < 0.05) ↓ Pain in IG at 15 weeks ↓ Fatigue in IG at 26 weeks follow-up ↓Fatigue in CON at 26 weeks follow-up **Inter-group** (*p* < 0.05) < Pain in IG compared with CG at 15 weeks.
Saeterbakken et al. (2017) [30]	**Initial/final sample size**: 34/31 withLow non-specific neck and shoulder pain. **Distribution** (*n*; % W; mean age ± SD): - **IG**: *n* = 10; 100% W; 41.0 ± 15.3 - **CG** A: *n* = 13; 100% W; 47.6 ± 11.9 - **CG B**: *n* = 11; 100% W; 50.3 ± 14.8	**Duration**: 10 weeks + 10 weeks follow-up **Volume**: 30 min/session Frequency: 2 sessions/week **IG**: Exercise: NW Intensity: 12–14 RPE **CG** A: Exercise: Strength training (exercises for the neck- and shoulder muscles with elastic bands) Intensity: Loads that allowed 12 repetitions (Bands provided 36 N, 72 N and 140 N resistance). CG B: Did not performed any exercise program.	- Pain (VAS 0–100)	**Adverse effects**: NR **Completion rate**: 91.2% **Dropouts**: IG = 1 CG A= 1 CG B= 1 **Reasons for dropout/exclusion**: NR	**Intra-group** (*p* < 0.05) ↓ Pain in IG post-intervention. ↓ Pain in IG at 10 weeks follow-up vs. baseline ↓ Pain in CG A post-intervention. **Inter-group** (*p* < 0.05) NO
Santoyo-Medina et al. (2023) [31]	**Initial/final sample size**: 57/52 with multiple sclerosis **Distribution** (*n*; % W; mean age ± SD): - **IG**: *n* = 29; 72.4% W; 51.1 ± 8.75 - **CG**: *n* = 28; 60.7% W; 52.89 ± 11.12	**Duration**: 10 weeks **Volume**: 60 min/session **Frequency**: 2 sessions/week **Intensity**: 60–70% HR reserve + resting HR **IG1**: Exercise: NW **CG**: Exercise: Cycloergometer and treadmill aerobic training	- Fatigue (Modified Fatigue Impact Scale)	**Adverse effects**: NR **Completion rate**: 91.22% **Dropouts**: IG: *n* = 2CG: *n* = 3 **Reasons for dropout/exclusion**: - 2 relapses - 1 mayor depression - 1 lived away. - 1 knee bursitis	**Intra-group** (*p* < 0.05) ↓ Fatigue in IG and CG after intervention **Inter-group** (*p* < 0.05) NR
Spafford et al. (2014) [23]	**Initial/final sample size**: 52/38 with intermittent claudication **Distribution** (*n*; % W; mean age ± SD): - **IG**: *n* = 28; 47,37% W; 65 ± 2 - **CG**: *n* = 24; 50,00% W; 65 ± 2	**Duration**: 12 weeks **Volume**: 30 min/session. **Frequency**: 3 sessions/week **IG**: Exercise: Unsupervised NW, indications provided weekly by phone call with a physical therapist. Intensity: “Normal pace” **CG**: They were instructed to walk.	- Pain (Borg scale CR-10) - Fatigue (Borg scale CR-10)	**Adverse effects**: NR **Completion rate**: 73.08% **Dropouts**: IG: *n* = 9 CG: *n* = 5Reasons **for dropout/exclusion**:Worsening of health status, did not meet the inclusion criteria after being randomized.	**Intra-group** (*p* < 0.05) NO **Inter-group** (*p* < 0.05) NO

<: Lower; ↑: Increment; ↓: Decrement; BPI-SF: Brief Pain Inventory—Short Form; CG: Control Group; FIQ: Fibromyalgia Impact Questionnaire; FSS: Fatigue Severity Scale; HR: Heart Rate; IG: Experimental Group; LBPRS: Low Back Pain Rating Scale; MFI: Multidimensional Fatigue Inventory; NMSS: Non-Motor Symptoms Scale; NO: Not Observed; NR: Not reported; NW: Nordic walking; PFS: Parkinson’s Fatigue Scale; RPE: Rating of Perceived Exertion; SD: Standard Deviation; VAS: Visual Analog Scale; W: Women.

**Table 2:** Results of the methodological evaluation of the included studies (PEDro scale).

First Author, Year	PEDro Item										Score	Quality
	1	2	3	4	5	6	7	8	9	10		
Acar et al. (2023) [34]	+	−	+	−	−	+	+	+	+	+	7/10	Good
Bjersing et al. (2012) [28]	+	−	+	−	−	−	−	−	+	+	4/10	Fair
Collins et al. (2003) [22]	+	+	+	−	−	−	+	−	+	+	6/10	Good
Cugusi et al. (2015) [24]	+	−	+	−	−	−	−	−	+	+	4/10	Fair
Deepa et al. (2023) [25]	+	−	+	−	−	−	+	+	+	+	6/10	Good
Fields et al. (2016) [27]	+	−	+	−	−	−	+	−	+	+	5/10	Fair
Figueiredo et al. (2013) [32]	+	−	+	−	−	+	+	−	+	+	6/10	Good
Granziera et al. (2020) [26]	+	−	+	−	−	+	+	−	+	+	6/10	Good
Hartvigsen et al. (2010) [15]	+	+	+	−	−	−	+	+	+	+	7/10	Good
Kocur et al. (2017) [33]	+	+	+	−	−	−	+	−	+	+	6/10	Good
Mannerkorpi et al. (2010) [29]	+	+	+	−	−	+	+	+	+	+	8/10	Good
Saeterbakken et al. (2017) [30]	−	−	+	−	−	−	+	−	+	+	4/10	Fair
Santoyo-Medina et al. (2023) [31]	+	−	+	−	−	+	+	+	+	+	7/10	Good
Spafford et al. (2014) [23]	+	−	+	−	−	−	−	−	+	+	4/10	Fair

1: Random allocation; 2: Concealed allocation; 3: Baseline comparability; 4: Blind participants; 5: Blind therapists; 6: Blind assessors; 7: Adequate follow-up; 8: Intention-to-treat analysis; 9: Between-group comparisons; 10: Point estimates and variability; +: Yes; −: No.

## Data Availability

No new data were created or analyzed in this study.
